# Meat Starter Culture Reduces *Aspergillus parasiticus* Production of Aflatoxins on Meat-Based and Salami Model Media

**DOI:** 10.3390/toxins16040173

**Published:** 2024-04-02

**Authors:** Iva Zahija Jazbec, Lea Demšar, Barbka Jeršek, Tomaž Polak

**Affiliations:** Department of Food Science and Technology, Biotechnical Faculty, University of Ljubljana, Jamnikarjeva 101, 1000 Ljubljana, Slovenia; lea.demsar@bf.uni-lj.si (L.D.); barbka.jersek@bf.uni-lj.si (B.J.); tomaz.polak@bf.uni-lj.si (T.P.)

**Keywords:** *Aspergillus parasiticus*, aflatoxins, meat starter culture, meat-based media, salami model media, dry-fermented meat products, food safety

## Abstract

There is great concern about the risk posed by the consumption of food contaminated with aflatoxins (AF), produced mostly by *Aspergillus* strains, that can also be found in dry-fermented meat products (DFMPs). The aim of this study was to investigate the inhibitory effect of meat starter culture (SC), frequently used for fermentation in the meat industry, on *A. parasiticus* growth and the production of aflatoxin B1 (AFB1), aflatoxin B2 (AFB2), aflatoxin G1 (AFG1), aflatoxin G2 (AFG2), and sterigmatocystin (STE) on different meat-based (CMA) and salami model (SM-G) media. Incubation was carried out under optimal conditions for fungal growth and under typical conditions for ripening of DFMPs for 21 days. Reversed-phase UPLC–MS/MS analysis was performed to determine mycotoxin production. SC reduced *A. parasiticus* growth more on CMA than on SM-G media. AFB1 formation was inhibited on both types of SC-containing media, although SC generally had a stronger inhibitory effect on AFB1 production on CMA than on SM-G. AFB1 and AFB2 were produced on CMA, while AFB1 dominated in SM-G, AFG1, and AFG2 were not detected in any media. The results show that SC inhibited AFB1 formation of *A. parasiticus* on SM-G media after 21 days of incubation under typical conditions for the production of DFMPs. These results indicate the necessity to investigate AF on natural matrices in an environment that is as similar as possible to real conditions in the production of DFMPs.

## 1. Introduction

The presence of mycotoxins in meat and meat products can be a result of fungal contamination during the production process, as fungi can originate from contaminated spices or other ingredients or from the production environment, especially in DFMPs. The origin of mycotoxins may be a carryover effect when mycotoxins are in the edible tissue of animals that have been fed contaminated feed [1,2,3,4]. The indigenous mold microbiota positively contributes to the unique characteristics of the final meat product, especially in traditional production [5,6]. The most frequently reported mycotoxins in cured and fermented meat products are ochratoxin A (OTA) and aflatoxins (AF), mainly aflatoxin B1 (AFB1) [1,6,7,8]. AFs are the most toxic and carcinogenic of the mycotoxins that contaminate food and feed, posing a global food safety hazard [9,10]. Among all mycotoxins, AFB1 is the most potent naturally occurring hepatocarcinogen that has been characterized so far. There is great concern about the risk of consuming food and feed contaminated by AFB1 [11,12]. AFs are produced by several species in *Aspergillus* section *Flavi*, and *Aspergillus flavus* and *A. parasiticus* are the main species commonly implicated as global contaminants of a wide variety of products [13]. Sterigmatocystin (STE) is a precursor to AFB1, and their chemical structures are similar. STE is a mycotoxin that should be considered because it can have a significant effect on human and animal health [14,15]. There is insufficient information on the incidence of STE and exposure to it by consumers, although the toxic effect of STE has already been demonstrated [16,17].

Traditionally fermented salami is generally fermented spontaneously. Starter cultures have been developed for use in modern meat fermentation processes to reduce batch-to-batch variation, ensure high product quality, and improve organoleptic properties. Starter cultures shorten fermentation times by rapidly acidifying the matrix to ensure low residual nitrate and nitrite levels in the final product and to standardize sensory properties [18]. The most widely used starter cultures in meat products are mixtures of lactic acid bacteria (LAB), staphylococci, and/or micrococci, and sometimes molds or yeasts [19]. LAB are biopreservative bacteria that ferment sugars and produce lactic and other acids, causing the pH to drop below 5.2 within 48 h at temperatures around 22–26 °C and relative humidity of 90–93% [20]. The resulting conditions reduce the growth of other microorganisms and also help extend the shelf life of meat products. The use of starters, especially LAB, as competitive microbiota in fermented meat products also provides another benefit: they outcompete spoilage microorganisms either by competing for nutrients, oxygen, and living space or by producing bacteriocins or other antagonistic compounds, such as organic acids, hydrogen peroxide, or enzymes [20]. LABs are also known to bind aflatoxins in different food matrices to some extent [21,22,23].

Fungal spoilage of foods represents a major cause of concern for food manufacturers. Numerous methods (chemical, biological, and physical) exist to prevent fungal growth and inhibit toxin production, but most of them are rarely used, primarily because they are expensive [21]. Meanwhile, these methods may cause the loss of nutritional value, altering the organoleptic characteristics of the products. In addition, traditionally used autochthonous food microorganisms may have potential in the prevention of mycotoxin-caused health effects [24]. As an alternative, biocontrol with the use of antagonistic microorganisms has been proposed for controlling toxigenic molds in foodstuffs, including fruits, wheat, dairy, and meat products [25,26,27]. The use of LAB to mitigate fungal spoilage of foods and feeds is a promising solution [28,29]. With increasing pressure from consumers regarding more natural food preservatives, LAB represents an ideal biocontrol agent due to its Generally Regarded As Safe (GRAS) status by the FDA and Qualified Presumption of Safety (QPS) status in the EU [28]. The role of LAB is not limited to inhibiting fungal growth, but some LAB strains can interact with mycotoxins, causing their inactivation or removal [30]. Three main mechanisms have been proposed for mycotoxin reduction by LAB: (i) binding of the mycotoxin to the bacterial cell wall components; (ii) degradation of the mycotoxins into less toxic substances by the LAB; and (iii) inhibition of mold growth or aflatoxin biosynthesis by specific bacterial metabolites [21,31].

A few studies have been conducted to screen LAB strains or microbial communities with antagonistic effects on mycotoxicogenic fungi in food-based media [31,32,33,34]. Even fewer studies have investigated the effect of LAB on the production of AF or OTA; the focus has been on examining the effect of temperature on growth and mycotoxin production in meat-based media [30]. It was shown that temperature might have a major impact on the production of mycotoxins [30,35,36].

The aim of this study was to use mixed meat starter culture on salami model media under typical conditions for the production of dry-fermented meat products to inhibit AFB1 formation by *A. parasiticus*. In order to achieve this, the investigation was carried out in three stages: (i) investigation of the growth and AF and STE production potential of *A. parasiticus* on meat-based and salami model media; (ii) the effect of meat starter culture; and (iii) the effect of different temperature regimes (constant optimal temperature for *A. parasiticus* growth vs. dynamic temperature typical of a ripening chamber) on the growth of *A. parasiticus* and the ability to reduce the formation of AF and STE.

## 2. Results and Discussion

*A. parasiticus* growth and the production of aflatoxins (AFB1, AFB2, AFG1, AFG2) and sterigmatocystin (STE) were assessed on meat-based (CMA) and salami model (SM-G) media at the optimal temperature for *A. parasiticus* growth and a dynamic temperature regime typical for salami ripening (Figure S1). To determine the effect of the meat starter culture (SC) on growth and mycotoxin formation, half of the culture media contained SC, and half did not. This experiment was carried out over a period of 21 days, as this is the typical duration for the production of dry fermented salami in the Mediterranean region. We took samples on days 2, 7, and 14 to obtain information about growth and mycotoxin production kinetics and on the last day of incubation (day 21). The influence of constant optimal temperature compared to dynamic temperature in the ripening chamber in interaction with SC on CMA and SM-G media was assessed.

### 2.1. Effect of Meat Starter Culture on A. parasiticus Growth and Mycotoxin Production on Meat-Based Media

**Figure 1:**
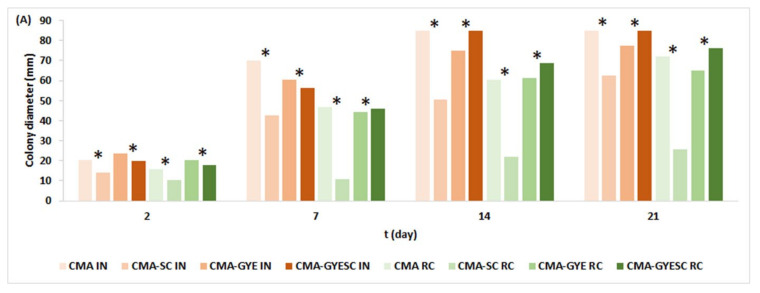

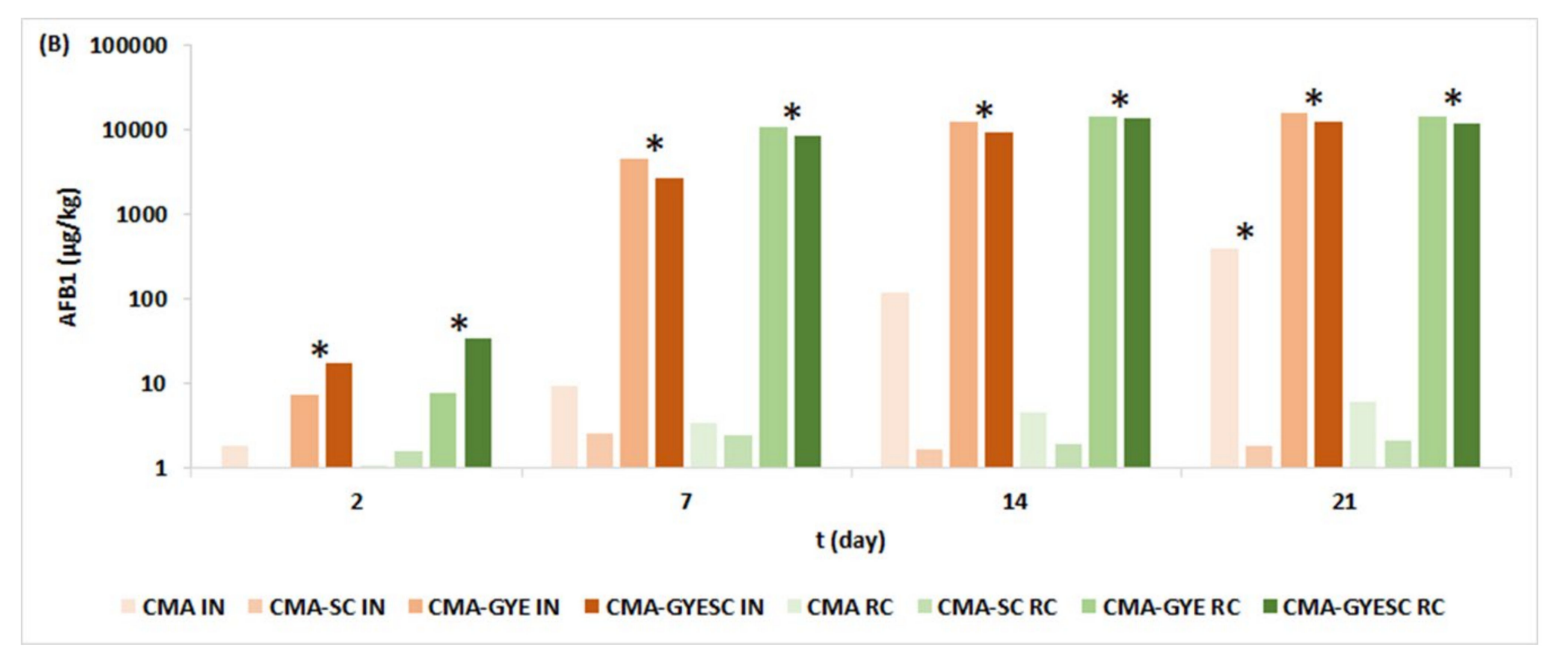
The effect of meat starter culture on the growth of *A. parasiticus* (**A**) and AFB1 production (**B**) on Cooked Meat Agar at 25 °C and at temperatures in the ripening chamber for 21 days. For abbreviations, see Section 4.2. IN: incubation at constant temperature of 25 °C; RC: temperatures typical for salami ripening presented in Figure S1 * represent statistically significant difference (*p* ≤ 0.05) between means of the same type of medium with SC and without SC incubated under same treatment (e.g., CMA-SC IN vs. CMA IN).

The addition of SC to CMA reduced the growth of *A. parasiticus*, as the colonies on CMA-SC media were smaller than those on CMA media, regardless of the temperature regime (Figure 1A). The reduced growth of *A. parasiticus* could have been due to direct competition (competitive exclusion) between *A. parasiticus* and the bacteria and yeast in SC for nutrients and space [37] or the production of hydrolytic enzymes, killer toxins, or volatile compounds [33].

When CMA was supplemented with glucose (G), yeast extract (YE), and meat starter culture (SC), the colonies of *A. parasiticus* on CMA-GYESC were again smaller than those on CMA-GYE after 7 days, but after 14 and 21 days, they were bigger on CMA-GYESC than on CMA-GYE at 25 °C (*p* ≤ 0.05) (Figure 1A). At lower temperatures, which are typical for the ripening chamber, colonies were already larger on CMA-GYESC than on CMA-GYE on day 7, and the same was observed after 14 and 21 days of incubation (*p* ≤ 0.05). SC inhibited the growth of *A. parasiticus* on CMA-GYSC for 7 days at 25 °C but not at lower temperatures in comparison with CMA-GYE; we assume that the reason may be that SC is less active at lower temperatures in the ripening chamber than at a constant temperature of 25 °C in an incubator.

The addition of G and YE to the CMA medium (CMA-GYESC) had a greater stimulative effect on the growth of *A. parasiticus* than the inhibitory effect of SC. Zahija et al. [38] already showed that the addition of G and YE to the CMA medium (CMA-GYE) increased the growth of *A. parasiticus* in comparison with its growth on CMA alone.

The mean colony diameter of *A. parasiticus* was almost twice as large at a constant temperature of 25 °C than at lower temperatures typical for a ripening chamber (Figure 1). This result was expected, as 25 °C is the optimal growth temperature for *A. parasiticus* [39]. Another reason could be the properties of the meat SC, which was not as active at the lower temperatures typical for the fermentation of meat products (17 or 16 °C) as it was at a constant temperature of 25 °C.

In parallel with the growth of *A. parasiticus*, AFB1 was also determined in all varieties of CMA media (Figure 1B). The addition of meat SC to the CMA medium reduced AFB1 production on CMA-SC and CMA-GYESC after 7, 14, and 21 days of incubation, irrespective of the temperature regime. There was greater production of AFB1 when G and YE were added to the medium (CMA-GYE) than on the medium without supplementation (CMA). AFB1 production was not influenced by incubation temperature (25 °C vs. lower temperatures typical for a ripening chamber) but by the composition of the medium (CMA vs. CMA-GYE). The addition of G and YE to CMA had a stimulating effect on AFB1 production. The highest yields of AFB1 were produced in CMA-GYE after 21 days at 25 °C (16,331 ± 360 µg/kg) and at temperatures typical for a ripening chamber (14,886 ± 319 µg/kg). The lowest AFB1 levels were observed on CMA-SC on all days of incubation (Figure 1B). The maximum average AFB1 production by *A. parasiticus* was recorded as 12,120 µg/kg in a control culture grown at 25 °C after 10 days in a study by Mateo et al. [30].

Reduced AFB1 formation on media containing SC was also found to be a characteristic of co-inoculation of *A. parasiticus* with *S. xylosus* on dry-cured ham-based agar [40], as *S. xylosus* is a component of the meat SC (Bactoferm SM-194, Chr. Hansen, Hørsholm, Denmark) used in this study. Peromingo et al. [27] reported that native *D. hansenii* had significant antagonistic activity against A. parasiticus AFB1 production in a meat model system, and *D. hansenii* is also a component of the mixed meat SC used in this study. Besides these two strains, the mixed meat SC also consists of *L. sakei*, *P. pentosaceus*, and *S. carnosus*. In a study by Mateo et al. [30], the level of AFB1 produced by *A. parasiticus* was higher in controls without LAB strains than in cultures in which LAB strains were present and incubated at the same temperature.

In a study by Delgado et al. [32], mycotoxin production in sausages was similar to fungal growth. *A. parasiticus* produced high AFB1 and AFG1 levels (86–115 µg/kg) in untreated sausages, at both 5 and 15 days of incubation. The combination of antifungal protein and *D. hansenii* had an inhibitory effect on *A. parasiticus* growth and aflatoxin production on sliced dry-fermented sausage.

AFB2 and STE formation during the 21-day growth of *A. parasiticus* on CMA media is shown in Table 1. Regardless of the incubation temperature (25 °C (IN) vs. temperature in ripening chamber (RC)), the influence of the medium composition was evident, as the formation of AFB2 and STE on CMA was very low compared to their formation on CMA-GYE. The highest yields of AFB2 were produced on CMA-GYE after 21 days in the incubator (2983 ± 65 µg/kg) and the ripening chamber (2805 ± 138 µg/kg). The highest STE yield was produced on CMA-GYE after 14 days in the incubator (16.75 ± 1.42 µg/kg) and 7 days in the ripening chamber (17.36 ± 1.19 µg/kg). It is evident that the meat SC reduced the formation of AFB2 and STE on the CMA-GYE SC media compared to the CMA-GYE media without it (Table 1) under both incubation regimes.

Concentrations of AFG1 and AFG2 were below LOD in all types of media and all types of treatments.

**Figure 2:**
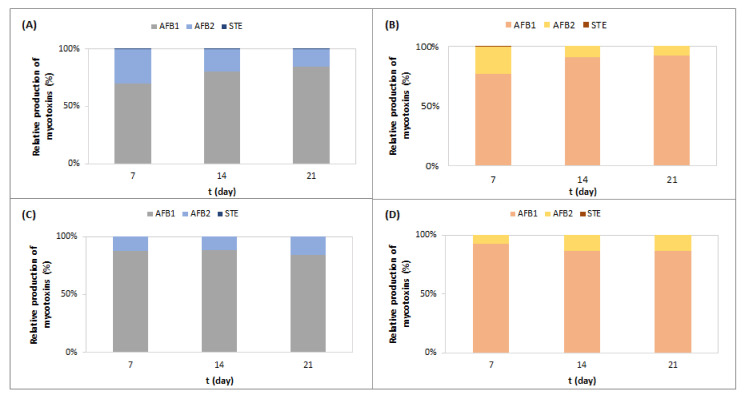
Relative production of AFB1, AFB2, and STE by *A. parasiticus* grown on CMA-GYE (**A**) and CMA-GYESC (**B**) at 25 °C (**A**,**B**) and on CMA-GYE (**C**) and CMA-GYESC (**D**) at temperatures typical for the ripening chamber (**C**,**D**) for 21 days. For abbreviations, see Section 4.2.

The comparison of the relative amounts of AFB1, AFB2, and STE produced by *A. parasiticus* on CMA-GYE and CMA-GYESC during 21 days of incubation at 25 °C shows that the AFB1 content increased over time, and the AFB2 content decreased, while the proportion of STE was less than 1%. At lower temperatures in the ripening chamber, AFB1 formation by *A. parasiticus* decreased over time; AFB2 formation increased, and the proportion of STE was less than 1% (Figure 2).

Growth conditions such as temperature, pH, water activity, and nutrient sources are known environmental factors that have a major influence on the production of secondary metabolites by fungi [41]. Simple sugars (e.g., glucose, sucrose, fructose) support AFB1 and AFB2 production by *A. flavus* and *A. parasiticus*, but more complex sugars (e.g., galactose, lactose) do not. Nitrogen sources used in growth media (nitrate or ammonia) have different effects on STE and aflatoxin production [42]. Nitrate has been shown to repress the synthesis of aflatoxin intermediates in *A. parasiticus* but enhance STE production in *A. nidulans*. CMA-GYE medium was supplemented with glucose (1%) and yeast extract (0.2%), as these components are also added to DFMP production to improve fermentation. In addition to vitamins and minerals, yeast extract also contains plenty of free amino acids and polypeptides and is an ideal nutrient growth medium for both laboratory and industrial microbial fermentation [43]. Polypeptides are the main nutrient factors that affect fungal growth and metabolism, but their composition and quality in yeast extract can vary depending on the raw material and process.

STE is a precursor to AFB1 and AFG1, which are produced as secondary metabolites in *A. flavus* and *A. parasiticus* through complex biosynthesis regulated by at least 53 genes, and although many investigators have studied them, there are still some gaps in our understanding; i.e., it is not known under which conditions *Aspergillus* fungi will stop AF production [44]. The International Agency for Research on Cancer classifies STE in group 2B, as it was shown to be hepatotoxic and nephrotoxic in animals. Fungi capable of producing STE are common food and feed contaminants with a consequent strong economic impact on the biotechnological, agricultural, and food industries [14,15]. Our results show that STE was also formed during 21-day incubation, with the highest peak on the 7th or 14th days, but only on media that was supplemented with glucose and yeast extract (CMA-GYE). Again, supplementation of the medium with a starter culture (CMA-GYESC) resulted in reduced formation of STE. It can be assumed that under given conditions, the majority of STE was converted to AFB1 [44].

### 2.2. Effect of Meat Starter Culture on A. parasiticus Growth and Mycotoxin Production on Salami Model Medium

**Figure 3:**
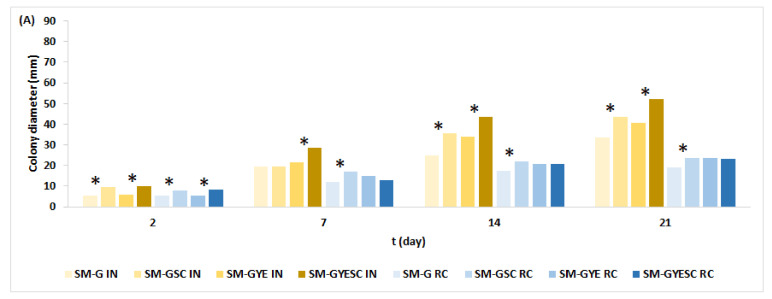

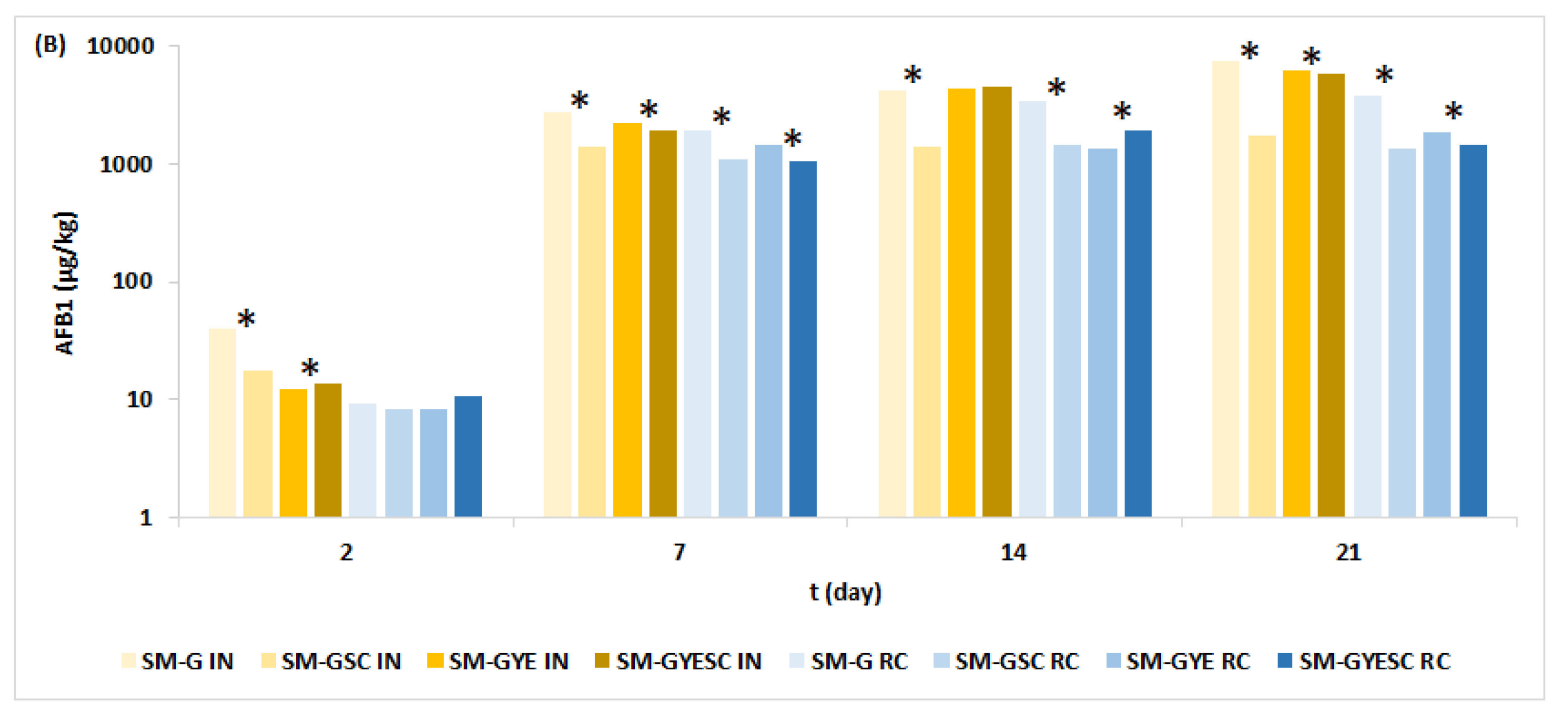
The effect of meat starter culture on the growth of *A. parasiticus* (**A**) and AFB1 production (**B**) on salami model medium at 25 °C and at ripening chamber temperatures for 21 days. For abbreviations, see Section 4.2. IN: incubation at constant temperature of 25 °C; RC: temperatures typical for salami ripening presented in Figure S1. * represents a statistically significant difference (*p* ≤ 0.05) between means of the same type of medium with SC and without SC incubated under the same treatment (e.g., CMA-SC IN vs. CMA IN).

The addition of meat SC to salami model (SM-G) media had an unexpected effect on the growth of *A. parasiticus* when incubated at 25 °C (Figure 3A). SC triggered the growth of *A. parasiticus* in most cases, and colonies were larger on SM-GSC and SM-GYESC than on SM-G and SM-GYE. SC was added to SM-G media according to the manufacturer’s instructions, in freeze-dried form during mixing, as is common in the meat industry, without prior growth (i.e., starter culture was not grown in the Man–Rogosa–Sharpe (MRS) broth for 24 h prior to inoculation into SM-G), and therefore, the SC cells might have been less active, or some of them were not able to grow. Meftah et al. [45] found that when SC was intentionally added to traditional sausage- and ham-based media as dead cells, *Penicillium nordicum* growth was increased in comparison to control media, but when actively growing SC cells were added to the media, the growth of *P. nordicum* was inhibited. It can be assumed that SM-G is less suitable for the initiation of SC growth than CMA and that adding SC can stimulate the growth of *A. parasiticus* on SM-G. The same stimulating effect of SC on the growth of *A. parasiticus* was observed on SM-GSC when incubated in the ripening chamber, but no effect of SC on the growth of *A. parasiticus* on SM-GYESC was detected. This indicates that some SC cell activity resulted in a slight inhibition of *A. parasiticus* growth after 7 days, but after 7, 14, and 21 days, the colony diameter on SM-GYESC and SM-GYE was the same (Figure 3A).

In general, the growth of *A. parasiticus* was affected by the type of media and SC (Figure 1 and Figure 3). SC showed a variable effect on fungal growth, having an effect in both directions: it stimulated and inhibited the growth of colonies, and these data corroborate the findings of [33]. SC was chosen because prior surveys showed that any study of biocontrol agents against mycotoxigenic fungi must involve the microbial community that is usually present in the matrix instead of isolated microorganisms [33].

**Figure 4:**
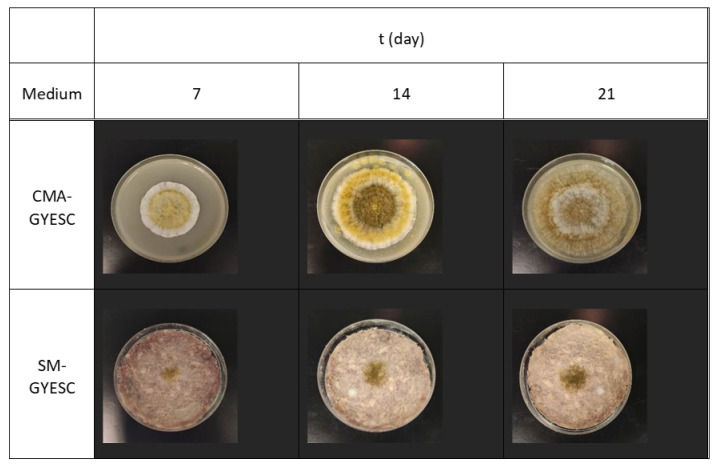
Colonies of *A. parasiticus* after 7, 14, and 21 days of incubation in the ripening chamber on meat-based media (CMA-GYESC) and salami model media (SM-GYESC).

The increased fungal growth on SM-GSC due to SC was unexpected under both temperature regimes since a reduction in fungal growth due to the starter effect on a meat-based media was previously reported [33,40,46,47]. This may be due to different experimental settings, such as the starter culture, components of the meat-based media, and casings. In our experiment, spore suspension was one-point inoculated directly onto the surface of meat-based media without the casing acting as a barrier (Figure 4).

In a study by Cebrián et al. [40], the pattern of development of *A. parasiticus* colonies on a dry-cured ham-based medium showed complete colonization of the Petri dish (5 cm diameter) over 11–14 days, depending on the incubation temperature (15, 20, 25 °C). In the same study, co-inoculation with *S. xylosus* had a marked influence on reducing the colony growth of *A. parasiticus*.

Colony color during 21 days in the ripening chamber was affected by the composition of the meat-based medium. Glucose in CMA led to the formation of green color on CMA-G, CMA-GYE, and CMA-GYESC. The addition of SC had more influence on colony diameter than colony color. Colony color on SM-G-based media was distinctively greener if SC was added, compared to SM without SC. The same effect on colony color was observed at 25 °C (Figure 4).

*A. parasiticus* production of AFB1 was reduced when the strain was grown on salami model (SM-G) media with added meat SC (SM-GSC, SM-GYESC) at 25 °C and at temperatures typical for the ripening chamber (Figure 3B). The exception was found only after 14 days on SM-GYE RC and SM-GYE IN. The addition of yeast extract (YE) to SM-G media, which already had glucose, had no effect or slightly reduced AFB1 production. The highest yields of AFB1 were produced on SM-G after 21 days at 25 °C (7348 ± 140 µg/kg) and at temperatures typical for the ripening chamber (3766 ± 137 µg/kg).

The influence of SC on mycotoxin production was found to be variable: *A. westerdijkiae* production of OTA was stimulated when commercial starter culture was added to industrial sausage-based medium (Meftah et al., 2018) and ham and traditional sausage media [45], but *P. nordicum* OTA production was inhibited on ham medium. Authors in [34] added *D. hansenii* and *L. buchneri* to fresh pork leg inoculated with *A. westerdijkiae*, and after 6 months of seasoning, OTA was not detected.

The effect of SC on reduced AFB2 production was observed on SM-GSC media incubated at 25 °C for 21 days and at temperatures typical to the ripening chamber for 14 days (Table 2). When yeast extract was added to the salami model medium (SM-GYESC), SC had no effect on AFB2 production after 21 days of incubation at temperatures typical for the ripening chamber. However, on SM-GYESC, SC stimulated AFB2 production after 14 and 21 days of incubation regardless of the temperature. STE production on SM-GSC and SM-GYESC media was generally higher than on SM-G and SM-GYE media, with two exceptions (day 7 on SM-GSC RC and SM-GYESC RC, and day 14 on SM-GYESC RC).

**Figure 5:**
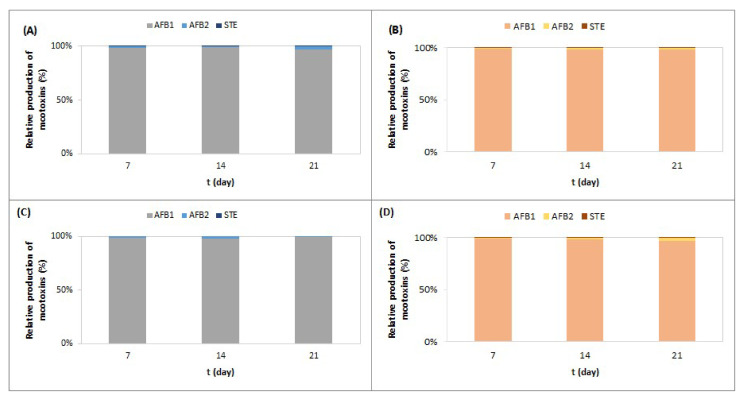
Relative production of AFB1, AFB2, and STC by *A. parasiticus* grown on SM-GYE (**A**) and SM-GYESC (**B**) at 25 °C (**A**,**B**) and on SM-GYE (**C**) and SM-GYESC (**D**) at temperatures typical for the ripening chamber (**C**,**D**) for 21 days. For abbreviations, see Section 4.2.

The comparison of AFB1, AFB2, and STE formation on SM-GYE and SM-GYESC shows that the AFB1 content was around 99% (Figure 5) under both temperature regimes.

A comparison of AFB1, AFB2, and STE formation on salami model media (Figure 5) shows that AFB1 dominated, while *A. parasiticus* produced AFB1 and AFB2 under the same conditions on CMA media (Figure 2). We assume that the composition of the salami model medium, which contained nitrite salt in addition to other raw materials (pork meat and back bacon), compared with cooked meat agar, as well as the preparation of the medium itself (without sterilization), influenced the dominant formation of AFB1. Nitrate suppresses AFB2 and STE formation in *A. parasiticus* [42]. The kinetics of AF and STE formation on CMA and SM-G differed (Figure 2 and Figure 5). Under conditions quite similar to the production of fast-fermented salami (RC), SC in SM-GYE reduced AFB1 and STE formation after 7 and 21 days of incubation (*p* ≤ 0.05).

The *A. parasiticus* strain used in this study produced STE, AFB1, and AFB2 but not AFG1 and AFG2 (≤LOD) on meat-based and salami model media. This finding is in agreement with Gallo et al. [36], although they used *A. flavus*.

The medium (substrate) and the incubation time influenced the colony diameter and the production kinetics of AF of the *A. parasiticus* strain, and this finding is consistent with our previous study [38]; the incubation regime also had an influence.

In general, *A. parasiticus* showed great potential for production of AFB1 on meat-based and salami model media and, therefore, should be considered as a significant potential contaminant of fermented meat products, but the addition of meat starter culture proved to have an inhibitory effect on AFB1 production. Further research is needed to determine any additional antagonistic or synergistic environmental effects that would further inhibit the formation of AFB1. The findings are consistent with our previous research [38], with a notable upgrade, as the conditions in the present study (salami model media incubated under conditions typical for a ripening chamber) are closer to industry practice.

### 2.3. Relation between A. parasiticus Growth and AFB1 Production on Meat-Based and Salami Model Media

In general, colonies of *A. parasiticus* were larger, and the production of AFB1 was greater on CMA than on SM-G media (Figure 2 and Figure 4). However, in general, SC had a greater inhibitory effect on AFB1 production on CMA than on SM-G (Table 3).

The results show a greater percentage of growth inhibition in CMA media than in SM-G media. Further, the addition of SC inhibited AFB1 formation of *A. parasiticus* on CMA and on CMA supplemented with G and YE under both incubation regimes, as well as on SM-G and SM-G supplemented with G and YE under both incubation regimes (except on SM-GYESC after 14 days). These conditions are close to the production conditions in the meat industry [48], and the results demonstrate the positive effect of the meat starter culture on controlling aflatoxins in fermented meat products. In a study by Mateo et al. [30], the minimum and maximum percentages of AF reduction compared to the controls in LAB treatment of *A. parasiticus* were 19.7% and 44.7%.

Meftah et al. [33] showed that inhibition of fungal growth should not lead to the assumption that mycotoxin production would also be repressed. Our results show that *A. parasiticus* growth was inhibited in CMA media but not in SM-G media in the presence of SC. We observed high concentrations of AFB1 in SM-G media on the last day of incubation under both temperature regimes (1300–7300 µg/kg), although the mean diameters of colonies were medium-sized (17–52 mm). Thus, our results are consistent with those of Meftah et al. [33].

## 3. Conclusions

The selected meat starter culture showed a good inhibitory effect on AF production on meat-based and salami model media. The incubation regime was found to be a parameter with a great deal of influence on AF kinetics in interactions with starter culture. The importance of using natural matrices, such as in the salami model media, in combination with the conditions present during fermentation in a ripening chamber was emphasized. The results show that the starter culture had more influence on reducing AF production than mold growth. The use of a meat starter culture consisting of different strains to control toxigenic substances in dry-fermented meat products (and, potentially, traditional meat products) should be considered a promising biocontrol approach. Further research focusing on conducting experiments on salami will be carried out in the future.

## 4. Materials and Methods

### 4.1. Microorganisms and Culture Conditions

Mixed meat starter culture (SC) (Bactoferm SM-194, Chr. Hansen) was used to assay its antifungal activity against *Aspergillus parasiticus* strain ŽMJ7. ŽMJ is a designation of the Culture Collection of Laboratory of Food Microbiology at the Department of Food Science, Biotechnical Faculty, University of Ljubljana, Slovenia). *A. parasiticus* was isolated from the ripening chamber of a traditional salami producer in Savinjska Valley, Slovenia. *A. parasiticus* ŽMJ7 was also used in our previous study [38] as it showed good AF production potential on meat-based medium. *A. parasiticus* was routinely grown on malt extract agar (MEA; Sigma-Aldrich Chemie GmbH, Steinheim, Germany) for 7 days at 25 °C. Spores from the surface of MEA plates were collected with a sterile inoculation loop and suspended in sterile agar (0.5%) with Tween 80 (0.1% *v*/*v*). The spores were quantified using a Thoma counting chamber (Brand, Wertheim, Germany), and the spore suspension was adjusted to 105 spores/mL and used as an inoculum.

Bactoferm SM-194 (Chr. Hansen) is a meat SC used for the production of traditional fermented sausage that has a short production time and provides Mediterranean flavor and a fast pH drop. SM-194 consists of *Lactobacillus sakei*, *Pediococcus pentosaceus*, *Staphylococcus xylosus*, *S. carnosus*, and *Debaryomyces hansenii*. Starter culture was used in selected media as an ingredient according to the manufacturer’s instructions (0.125 g/kg).

### 4.2. Culture Media Preparation

In the experimental part, four types of meat-based media designated as cooked meat agar (CMA) and four types of salami model (SM-G) media were used to obtain similar conditions to what is typical for the production of DFMP.

Basic CMA was prepared by boiling veal bones (4 kg) in distilled water (4 L) for 4 h, which was concentrated to 6% dry weight during cooking [38]. The mixture was filtered through a double layer of muslin, and 20 g/L bacteriological agar was added prior to sterilization. CMA medium was autoclaved at 121 °C (103 kPa) for 20 min, then cooled down to 45–50 °C and poured (20 mL) into 90 mm diameter Petri dishes or supplemented with different supplements prior to pouring into Petri dishes.

For the cooked meat agar with Bactoferm SM-194 starter culture (SC) (CMA-SC), SC was added to the CMA medium after the medium was sterilized and cooled to 45–50 °C.

For the cooked meat agar with glucose (G) and yeast extract (YE) (CMA-GYE), the CMA medium was supplemented with 1% glucose (D-(+)-glucose; Sigma-Aldrich Chemie GmbH, 50-99-7) and 0.2% YE (Sigma-Aldrich Chemie GmbH) prior to sterilization.

For the meat agar cooked with glucose, yeast extract, and starter culture (CMA-GYESC), the medium was prepared as CMA-GYE, and after being sterilized and cooled down to 45–50 °C, SC was added.

The basic salami model with glucose (SM-G) was prepared by mixing pork (triceps brachii, 82%) and back bacon (18%) by using a cutter (Krämer & Grebe, Biendenkopf-Wallau, Germany). The other ingredients (nitrite salt (2.4%), a mixture of 0.6% sodium nitrite and 99.4% NaCl, and glucose (1%)) were added and mixed until the required temperature (1 °C) was reached. The medium was not autoclaved. The amounts of ingredients were calculated in relation to the raw material weight.

For the salami model with glucose and starter culture (SM-GSC), SC was added to the basic SM-G medium.

For the salami model with glucose and yeast extract (SM-GYE), the basic SM-G medium was supplemented with 0.2% YE.

For the salami model with glucose, yeast extract, and starter culture (SM-GYESC), the basic SM-G was supplemented with 0.2% YE and SC.

In these experiments, 30 g of each salami model medium was weighed into 90 mm diameter Petri dishes and used immediately.

### 4.3. Experimental Conditions and Design of Experimental Work

All media were centrally inoculated with 10 μL of *A. parasiticus* spore suspension (10^5^ spores/mL). One set of media was incubated in the dark at 25 °C and constant relative humidity (97%) for up to 21 days (first incubation regime), and the other set was incubated in a ripening chamber (Memmert HPP260 constant climate chamber; Memmert, Schwabach, Germany) for up to 21 days (second incubation regime). The ripening protocol consisted of an eight-phase process until the end of the experiment (21 days): 12 h at 23 °C and RH 90%; 24 h at 22 °C and RH 88%; 24 h at 21 °C and RH 85%; 24 h at 20 °C and RH 80%; 24 h at 19 °C and RH 80%; 24 h at 18 °C and RH 80%; 24 h at 17 °C and RH 80%; and 16 °C and RH 75% (Figure S1). The first regime was selected as the optimal temperature for *A. parasiticus* growth, and the second represents temperature and relative humidity conditions during meat product fermentation in the ripening chamber. All experiments were performed with 3 replicates per treatment.

During the 21-day incubation, *A. parasiticus* growth was measured, and samples were collected for AF and STE analysis after 2, 7, 14, and 21 days (Figure S1) and frozen at −80 °C until further analysis. We analyzed growth and mycotoxin production at four points to obtain the kinetics of growth and mycotoxin production in the time period typical for dry-fermented meat production.

### 4.4. Assessment of A. parasiticus Growth

*A. parasiticus* growth was determined on days 2, 7, 14, and 21 by measuring the diameter of colonies in two directions at right angles to each other. All colonies were also photographed. These data were utilized to determine the mean colony diameter as the measure of *A. parasiticus* growth.

Inhibition of *A. parasiticus* growth (ING) by starter culture (SC) was calculated as follows: ING (%) = [(D1 − D2)/D1] × 100, where D1 represents the average diameter of *A. parasiticus* on medium without the addition of starter culture (CMA, CMA-GYE, SM-G, and SM-GYE) and D2 is the average diameter of *A. parasiticus* on the same medium with the addition of starter culture (CMA-SC, CMA-GYESC, SM-GSC, and SM-GYESC) [34].

### 4.5. Extraction and Quantification of Aflatoxins and Sterigmatocystin

#### 4.5.1. Sampling and Mycotoxin Extraction

To determine AF and STE production, samples were collected on days 2, 7, 14, and 21. AF and STE were extracted using the methodology described by Zahija et al. (2023) [38]. Briefly, the entire medium was extracted with 35 mL of 80% acetonitrile with 0.5% HCOOH added to the medium in an ultrasonic bath (Branson 3510, Brookfield, CT, USA) for 15 min. Afterward, the mixture was passed through filter paper (Sartorius 388) and then hand-shaken. Then, the samples were purified using Phree™ phospholipid removal tubes (8B-S133-TAK, Phenomenex, Torrance, CA, USA) and passed through a 0.2 μm nylon filter (AF0-0499, Phenomenex, Torrance, CA, USA) and transferred into HPLC vials. Samples were analyzed in duplicate for the presence of AFB1, AFB2, AFG1, AFG2, and STE.

#### 4.5.2. Quantification of AF and STE by UPLC–MS/MS Analysis

UPLC–MS/MS analyses were performed with an ACQUITY™ UPLC™ H-Class PLUS (Waters, Milford, MA, USA) system consisting of a thermostated Sample Manager–FTN, a thermostated column compartment, and a quaternary solvent manager. The UPLC system was coupled with a Xevo TQ-S micro triple quadrupole mass spectrometer (Waters). The instrument data were collected and processed using MassLynx software (V4.2 SCN1017, 2020; Waters). Separation of analytes was performed using a reversed-phased ACQUITY Premier UPLC^®^ CSH C18 column (100 mm × 2.1 mm, 1.7 μm). The conditions were as follows: column temperature, 40 °C; injection volume, 2 μL; flow rate of mobile phase, 400 μL/min. The mobile phases were composed of two eluents, both containing 0.5% acetic acid and 0.1% formic acid; eluent A was 1 mM ammonium acetate in water, and eluent B was acetonitrile (720007377, September 2021; Waters). The mobile phase gradient was programmed as follows: 0.00–0.70 min, 5% B; 0.70–6.50 min, 5–50% B; 6.50–9.50 min, 50–100% B; 9.50–12.50 min, 100% B; 12.50–12.60 min, 100–5% B; and 12.60–14.00 min, 5% B. The mass spectrometer was operated in positive ionization mode (ESI+) with the following operating conditions: electrospray capillary voltage, 0.75 kV; cone voltage, 30 V; extractor voltage, 2 V; source temperature, 150 °C; desolvation temperature, 600 °C; cone gas flow rate, 50 L/h; desolvation gas flow rate, 1000 L/h; and collision energy, 20 eV. AF and STE were identified by comparing the retention time and mass spectrometric data, then quantified according to peak areas against previously determined calibration curves. The results of the validation process and calibration parameters for different media are presented in Table 4.

Inhibition of *A. parasiticus* AFB1 formation by starter culture (INA) was calculated as INA (%) = [(AF1 − AF2)/AF1] × 100, where AF1 represents the average AFB1 production on medium without the addition of starter culture (CMA, CMA-GYE, SM-G, and SM-GYE), and AF2 is the average AFB1 production on the same medium with the addition of starter culture (CMA-SC, CMA-GYESC, SM-GSC, and SM-GYESC) [34].

### 4.6. Statistical Analysis

This statistical analysis was carried out using IBM SPSS v.22 software (IBM, Armonk, NY, USA). Once the dependent variables (colony diameter, AF, and STE content) and independent variables (days of sampling, different batches) for the analysis were determined, a study of the normality of the data populations was carried out using the Shapiro–Wilk test. As the results showed a non-parametric distribution, the Kruskal–Wallis test was performed. Since differences between groups (in terms of days or medium and incubation conditions) could not be evaluated with a non-parametric test, ANOVA was also performed, and the treatment was upgraded with a post hoc Duncan test of multiple comparisons. Statistical significance was established at *p* ≤ 0.05.

## Figures and Tables

**Table 1:** Mycotoxin production (mean ± SD µg/kg) by *A. parasiticus* grown on cooked meat agar media at 25 °C and at temperatures in the ripening chamber for 21 days.

Medium and Incubation Conditions	AFB2 (µg/kg)					STE (µg/kg)				
	2 Days	7 Days	14 Days	21 Days	KW (P)	2 Days	7 Days	14 Days	21 Days	KW (P)
CMA IN	0.06 ^c^ ± 0.07	0.19 ^b^ ± 0.02	0.26 ^ab^ ± 0.09	0.29 ^a^ ± 0.09	0.003	0.03 ^c^ ± 0.02	0.15 ^b^ ± 0.03	0.16 ^b^ ± 0.03	0.22 ^a^ ± 0.06	0.001
CMA-SC IN	0.17 ^a^ ± 0.02	0.22 ^a^ ± 0.07	0.17 ^a^ ± 0.02	0.17 ^a^ ± 0.04	0.236	0 ^d^ ± 0	0.02 ^a^ ± 0.00	0.05 ^a^ ± 0.01	0.01 ^c^ ± 0.01	≤0.001
CMA-GYE IN	0.57 ^c^ ± 0.16	1984.25 ^b,^* ± 68.8	2966.39 ^a,^* ± 76.67	2983.54 ^a,^* ± 65.91	≤0.001	0.07 ^d,^* ± 0.02	13.83 ^b,^* ± 1.29	16.75 ^a,^* ± 1.42	4.83 ^c,^* ± 0.58	≤0.001
CMA-GYESC IN	0.45 ^c^ ± 0.21	798.54 ^b,^* ± 77.01	978.86 ^a,^* ± 116.34	1037.78 ^a,^* ± 91.53	≤0.001	0.22 ^d,^* ± 0.04	3.84 ^a,^* ± 0.58	1.47 ^c,^* ± 0.16	3.15 ^b,^* ± 0.41	≤0.001
CMA RC	0.09 ^c^ ± 0.06	0.12 ^c^ ± 0.06	0.19 ^ab^ ± 0.12	0.26 ^a^ ± 0.11	0.027	0 ^d^ ± 0	0.15 ^a^ ± 0.02	0.04^c^ ± 0.01	0.11 ^d^ ± 0.01	≤0.001
CMA-SC RC	0.17 ^b^ ± 0.02	0.17 ^b^ ± 0.03	0.19 ^b^ ± 0.03	0.23 ^a^ ± 0.05	0.070	0 ^c^ ± 0	0.03 ^a^ ± 0.01	0.01 ^b^ ± 0.00	0.00 ^c^ ± 0.00	≤0.001
CMA-GYE RC	0.24 ^d,^* ± 0.08	1546.95 ^c,^* ± 113.30	1867.97 ^b,^* ± 85.96	2805.11 ^a,^* ± 138.82	≤0.001	0.15 ^d,^* ± 0.13	17.36 ^a,^* ± 1.19	10.25 ^b,^* ± 1.29	5.16 ^c,^* ± 0.92	≤0.001
CMA-GYESC RC	0.61 ^d,^* ± 0.23	688.88 ^c,^* ± 101.99	2131.86 ^b,^* ± 119.28	1855.87 ^a,^* ± 92.47	≤0.001	0.38 ^d,^* ± 0.06	2.70 ^b,^* ± 0.63	8.65 ^a,^* ± 0.40	2.09 ^c,^* ± 0.18	≤0.001

KW—Kruskal–Wallis test; P—statistical significance. For abbreviations, see Section 4.2. IN: incubation at constant temperature of 25 °C; RC: temperatures typical for salami ripening presented in Figure S1. a–d: data with different superscript letters within row differ significantly (*p* ≤ 0.05). * represents statistically significant difference (*p* ≤ 0.05) between same type of media and the same incubation treatment incubated with and without SC (e.g., CMA-SC IN vs. CMA IN).

**Table 2:** Mycotoxin production (mean ± SD µg/kg) by *A. parasiticus* grown on salami model media at 25 °C and at ripening chamber temperatures for 21 days.

Medium and Incubation Conditions	AFB2 (µg/kg)					STE (µg/kg)				
	2 Days	7 Days	14 Days	21 Days	KW (P)	2 Days	7 Days	14 Days	21 Days	KW (P)
SM-G IN	0 ^d^ ± 0	17.78 ^c^ ± 0.90	59.48 ^b^ ± 0.90	75.55 ^a,^* ± 1.55	≤0.001	0.57 ^d^ ± 0.13	1.16 ^c,^* ± 0.27	2.18 ^b^ ± 0.58	2.31 ^a^ ± 0.48	≤0.001
SM-GSC IN	0 ^d^ ± 0	11.10 ^c^ ± 0.71	17.52 ^b^ ± 1.29	44.21 ^a,^* ± 1.66	≤0.001	0.55 ^c^ ± 0.06	2.37 ^b,^* ± 0.14	2.79 ^a^ ± 0.17	2.66 ^a^ ± 0.37	0.001
SM-GYE IN	0 ^d^ ± 0	28.69 ^c^ ± 1.35	39.32 ^b^ ± 0.52	64.40 ^a,^* ± 0.90	≤0.001	0.56 ^b,^* ± 0.07	1.59 ^a,^* ± 0.29	1.61 ^a,^* ± 0.45	1.44 ^a,^* ± 0.22	0.003
SM-GYESC IN	0 ^d^ ± 0	16.99 ^c^ ± 1.60	61.89 ^b^ ± 1.99	96.84 ^a,^* ± 5.44	≤0.001	0.28 ^d,^* ± 0.16	2.64 ^b,^* ± 0.73	4.10 ^a,^* ± 0.72	2.63 ^b,^* ± 0.21	≤0.001
SM-G RC	0 ^d^ ± 0	9.60 ^c^ ± 0.73	36.77 ^b^ ± 1.64	21.56 ^a^ ± 1.55	≤0.001	0.30^d^ ±0.18	3.12 ^a^ ± 0.28	1.17 ^c,^* ± 0.12	1.79 ^b,^* ± 0.10	≤0.001
SM-GSC RC	0 ^d^ ± 0	4.95 ^c^ ± 0.31	14.44 ^b^ ± 2.90	21.18 ^a^ ± 1.58	≤0.001	0.29 ^c^ ± 0.17	2.67 ^b^ ± 0.17	2.78 ^b,^* ± 0.35	3.33 ^a,^* ± 0.65	0.001
SM-GYE RC	0 ^d^ ± 0	14.77 ^b^ ± 0.58	25.12 ^c^ ± 1.62	14.73 ^b^ ± 1.11	≤0.001	0.27 ^d^ ± 0.16	4.74 ^a,^* ± 0.25	1.72 ^c^ ± 0.15	2.15 ^b^ ± 0.16	≤0.001
SM-GYESC RC	0 ^d^ ± 0	6.01 ^c^ ± 0.91	31.56 ^b^ ± 2.66	48.41 ^a^ ± 5.45	≤0.001	0.25 ^c^ ± 0.22	3.08 ^a,^* ± 0.54	2.00 ^b^ ± 0.22	1.83 ^b^ ± 0.41	≤0.001

KW—Kruskal_Wallis test; P—statistical significance. For abbreviations, see Section 4.2. IN: incubation at constant temperature of 25 °C; RC: temperatures typical for salami ripening presented in Figure S1. a–d data with different superscript letters within row differ significantly (*p* ≤ 0.05). * represents statistically significant difference (*p* ≤ 0.05) between same type of media and the same incubation treatment incubated with and without SC (e.g., CMA-SC IN vs. CMA IN).

**Table 3:** Inhibition (%) of *A. parasiticus* growth (ING) and AFB1 formation (PIA) in meat-based and salami model media with addition of starter culture at 25 °C and in ripening chamber temperatures for 21 days compared to media without starter culture.

T	Inhibition of Growth (% ING)/Inhibition of AFB1 Formation (% INA)							
	Incubator at 25 °C		Ripening Chamber		Incubator at 25 °C		Ripening Chamber	
t (Day)	CMA-SC	CMA-GYESC	CMA-SC	CMA-GYESC	SM-GSC	SM-GYESC	SM-GSC	SM-GYESC
7	38.8/72.3	6.6/42.7	76.9/29.8	0/21.2	0/49.2	0/14.3	0/42.7	12.3/25.5
14	40.8/98.6	0/21.5	63.4/57.7	0/4.6	0/66.3	0/0	0/57.6	0/0
21	26.3/99.5	0/20.8	64.7/65.2	0/19.0	0/76.2	0/5.8	0/63.8	1.4/20.6

For abbreviations, see Section 4.2. IN: incubation at constant temperature of 25 °C; RC: temperatures typical for salami ripening presented in Figure S1. 0: no inhibition.

**Table 4:** UPLC-MS/MS conditions for investigating the levels of aflatoxins (AFs): B1 (AFB1); B2 (AFB2); G1 (AFG1); G2 (AFG2); and sterigmatocystin (STE) in spiked samples *.

Mycotoxin ID	Retention Time (min)	Precursor Ion	Cone Voltage (V)	Product Ion	Collision Energy (eV)	Recovery (%)	LOD (pg/column)	LOQ (pg/column)
AFB1	6.33	313.0	21	284.9 ^a^ 241.1 ^b^	38 22	93.2 ± 0.8	35	100
AFB2	6.01	315.0	21	287.1 ^a^ 259.1 ^b^	38 22	90.5 ± 2.3	67	220
AFG1	5.99	328.0	21	311.1 ^a^ 243.1 ^b^	38 22	92.3 ± 2.1	52	160
AFG2	5.66	330.0	21	313.1 ^a^ 245.1 ^b^	38 22	91.8 ± 3.3	73	253
STE	8.64	325.0	21	281.0 ^a^ 210.0 ^b^	38 22	89.3 ± 1.7	70	220

^a^ Transitions used for quantification. ^b^ Transitions used for confirmation. * Aflatoxin Mix 4 solution 34036 (Supelco, Bellefonte, PA, USA): AFB1 and AFG1: 0.1–100 ng mL^−1^; AFB2 and AFG2: 0.1–30 ng mL^−1^; STE 2–50 ng mL^−1^.

## Data Availability

Data are contained within the article.

## Supplementary Materials

The following supporting information can be downloaded at: https://www.mdpi.com/article/10.3390/toxins16040173/s1, Figure S1: Temperature and relative humidity during the 21 day incubation of media in the ripen-ing chamber (RC) and in the incubator (IN); Table S1: Aflatoxin B1 production (mean ± SD µg/kg) and colony diameter (mean ± SD mm) by *A. parasiticus* grown on cooked meat agar media at 25 °C and at temperatures in the ripening chamber for 21 days; Table S2: Aflatoxin B1 production (mean ± SD µg/kg) and colony diameter (mean ± SD mm) by *A. parasiticus* grown on salami model media at 25 °C and at ripening chamber temperatures during 21 days.
