# Supplementary material for: Meat Starter Culture Reduces Aspergillus parasiticus Production of Aflatoxins on Meat-Based and Salami Model Media
Source: Toxins (Basel). 2024 Apr 2;16(4):173. doi: 10.3390/toxins16040173 (PMC11053754; doi:10.3390/toxins16040173)
Supplement: Supplementary file 1 [file toxins-16-00173-s001.zip › toxins-2886941-supplementary.pdf]

# Supplementary Materials: Meat Starter Culture Reduces *Aspergillus parasiticus* Production of Aflatoxins on Meat-Based and Salami Model Media

**Iva Zahija Jazbec, Lea Demšar, Barbka Jeršek and Tomaž Polak**

**Table S1.** Aflatoxin B1 production (mean ± SD μg/kg) and colony diameter (mean ± SD mm) by *A. parasiticus* grown on cooked meat agar media at 25 °C and at temperatures in the ripening chamber for 21 days.

| Medium and incubation conditions | AFB1 (μg/kg) | | | | | COLONY DIAMETER (mm) | | | | |
|---|---|---|---|---|---|---|---|---|---|---|
| | 2 days | 7 days | 14 days | 21 days | KW (P) | 2 days | 7 days | 14 days | 21 days | KW (P) |
| CMA IN | $1.86^{d}$ ±0.17 | $9.18^{c}$ ±0.44 | $116.94^{b}$ ±2.79 | $397.89^{a,*}$ ±5.68 | ≤ 0.001 | $20.50^{c,*}$ ±0.34 | $70.00^{b,*}$ ±0.26 | $85.00^{a,*}$ ±0.00 | $85.00^{a,*}$ ±0.00 | ≤ 0.001 |
| CMA-SC IN | $1.03^{c}$ ±0.15 | $2.54^{a}$ ±0.29 | $1.67^{b}$ ±0.38 | $1.86^{b,*}$ ±0.43 | 0.034 | $14.17^{d,*}$ ±0.17 | $42.83^{c,*}$ ±0.31 | $50.33^{b,*}$ ±0.21 | $62.67^{a,*}$ ±0.84 | ≤ 0.001 |
| CMA-GYE IN | $7.29^{d,*}$ ±0.38 | $4652.73^{c,*}$ ±73.11 | $12356.73^{b,*}$ ±104.63 | $16330.81^{a,*}$ ±146.72 | ≤ 0.001 | $23.50^{d,*}$ ±0.22 | $60.50^{c,*}$ ±0.22 | $75.00^{b,*}$ ±0.20 | $77.50^{a,*}$ ±0.22 | ≤ 0.001 |
| CMA-GYESC IN | $17.16^{d,*}$ ±2.60 | $2667.08^{c,*}$ ±132.34 | $9696.74^{b,*}$ ±158.44 | $12939.97^{a,*}$ ±120.91 | ≤ 0.001 | $19.83^{c,*}$ ±0.17 | $56.50^{b,*}$ ±0.56 | $85.00^{a,*}$ ±0.00 | $85.00^{a,*}$ ±0.00 | ≤ 0.001 |
| CMA RC | $1.09^{d}$ ±0.20 | $3.46^{c}$ ±0.18 | $4.56^{b}$ ±0.13 | $6.21^{a}$ ±0.08 | ≤ 0.001 | $15.83^{d,*}$ ±0.31 | $46.83^{c,*}$ ±0.31 | $60.50^{b,*}$ ±0.56 | $72.17^{a}$ ±0.40 | ≤ 0.001 |
| CMA-SC RC | $1.62^{a}$ ±0.27 | $2.43^{a}$ ±0.35 | $1.93^{a}$ ±0.40 | $2.16^{a}$ ±0.42 | 0.365 | $10.50^{c,*}$ ±0.34 | $10.83^{c,*}$ ±0.31 | $22.17^{b,*}$ ±0.31 | $25.50^{a}$ ±0.50 | ≤ 0.001 |
| CMA-GYE RC | $7.86^{c,*}$ ±0.77 | $10789.54^{b,*}$ ±124.84 | $14536.24^{a,*}$ ±193.29 | $14886.08^{a,*}$ ±130.27 | ≤ 0.001 | $20.50^{d,*}$ ±0.34 | $44.50^{c,*}$ ±0.43 | $61.17^{b,*}$ ±0.40 | $65.00^{a}$ ±0.37 | ≤ 0.001 |
| CMA-GYESC RC | $34.52^{d,*}$ ±5.23 | $8496.85^{c,*}$ ±756.01 | $13873.48^{a,*}$ ±284.95 | $12065.03^{b,*}$ ±239.55 | ≤ 0.001 | $18.00^{d,*}$ ±0.26 | $46.00^{c,*}$ ±0.37 | $68.83^{b,*}$ ±0.40 | $76.17^{a}$ ±0.40 | ≤ 0.001 |

KW – Kruskal-Wallis test; P - statistical significance. For abbreviations, see the paragraph 4.2 Culture media preparation. IN: incubation at constant temperature of 25 °C; RC: temperatures typical for salami ripening presented in Fig. S1. a-d: data with different superscript letters within row differ significantly ($p \leq 0.05$). * represents statistically significant difference between same type of media and the same incubation treatment incubated with and without SC ($p \leq 0.05$).

**Table S2.** Aflatoxin B1 production (mean ± SD μg/kg) and colony diameter (mean ± SD mm) by *A. parasiticus* grown on salami model media at 25 °C and at ripening chamber temperatures during 21 days.

| Medium and incubation conditions | AFB1 (μg/kg) | | | | | COLONY DIAMETER (mm) | | | | |
|---|---|---|---|---|---|---|---|---|---|---|
| | 2 days | 7 days | 14 days | 21 days | KW (P) | 2 days | 7 days | 14 days | 21 days | KW (P) |
| SM-G IN | 40.21$^{d,*}$ ±6.11 | 2726,36$^{c,*}$ ±46.23 | 4148,87$^{b,*}$ ±25.44 | 7357.69$^{a,*}$ ±58.02 | ≤ 0.001 | 5.33$^{d,*}$ ±0.21 | 19.67$^{c}$ ±0.33 | 24.67$^{b,*}$ ±0.49 | 33.50$^{a,*}$ ±0.56 | ≤ 0.001 |
| SM-GSC IN | 17.33$^{c,*}$ ±0.71 | 1383.89$^{b,*}$ ±56.95 | 1397.64$^{b,*}$ ±77.58 | 1748,36$^{a,*}$ ±36.71 | ≤ 0.001 | 9.50$^{d,*}$ ±0.34 | 19.67$^{c,*}$ ±0.49 | 35.83$^{b,*}$ ±0.31 | 43.67$^{a,*}$ ±0.49 | ≤ 0.001 |
| SM-GYE IN | 12.15$^{d}$ ±0.88 | 2226.37$^{c,*}$ ±107.82 | 4405.24$^{b}$ ±115.53 | 6169.45$^{a,*}$ ±112.82 | ≤ 0.001 | 5.83$^{d,*}$ ±0.31 | 21.50$^{c,*}$ ±0.43 | 34.00$^{b,*}$ ±0.37 | 40.67$^{a,*}$ ±0.33 | ≤ 0.001 |
| SM-GYESC IN | 13.58$^{d}$ ±1.26 | 1907.98$^{c,*}$ ±186.03 | 4437.13$^{b}$ ±135.56 | 5810.05$^{a,*}$ ±50.95 | ≤ 0.001 | 9.83$^{d,*}$ ±0.31 | 28.50$^{c,*}$ ±0.34 | 43.67$^{b,*}$ ±0.49 | 52.00$^{a,*}$ ±0.26 | ≤ 0.001 |
| SM-G RC | 9.18$^{c}$ ±0.29 | 1901.59$^{b,*}$ ±59.60 | 3394.03$^{b,*}$ ±73.95 | 3766.93$^{a,*}$ ±88.42 | ≤ 0.001 | 5.33$^{d,*}$ ±0.21 | 12.00$^{c,*}$ ±0.26 | 17.33$^{b,*}$ ±0.80 | 19.17$^{a,*}$ ±0.40 | ≤ 0.001 |
| SM-GSC RC | 8.31$^{c}$ ±0.28 | 1089.05$^{b,*}$ ±19.47 | 1439.15$^{a,*}$ ±56.76 | 1362.30$^{a,*}$ ±27.49 | ≤ 0.001 | 7.83$^{d,*}$ ±0.31 | 17.17$^{c,*}$ ±0.79 | 21.83$^{b,*}$ ±0.17 | 23.50$^{a,*}$ ±0.34 | ≤ 0.001 |
| SM-GYE RC | 8.37$^{d}$ ±0.32 | 1427.43$^{b,*}$ ±25.95 | 1361.61$^{c,*}$ ±41.38 | 1826.73$^{a,*}$ ±73.88 | ≤ 0.001 | 5.33$^{d,*}$ ±0.21 | 14.83$^{c,*}$ ±0.31 | 20.83$^{b,*}$ ±0.31 | 23.50$^{a,*}$ ±0.34 | ≤ 0.001 |
| SM-GYESC RC | 10.51$^{d}$ ±0.73 | 1063.36$^{c,*}$ ±22.26 | 1941.55$^{a,*}$ ±100.78 | 1450.81$^{b,*}$ ±51.93 | ≤ 0.001 | 8.50$^{d,*}$ ±0.22 | 13.00$^{c,*}$ ±0.26 | 20.83$^{b}$ ±0.31 | 23.17$^{a}$ ±0.40 | ≤ 0.001 |

KW – Kruskal-Wallis test; P - statistical significance. For abbreviations, see the paragraph 4.2 Culture media preparation. IN: incubation at constant temperature of 25 °C; RC: temperatures typical for salami ripening presented in Fig. S1. a-d: data with different superscript letters within row differ significantly ($p \leq 0.05$). * represents statistically significant difference between same type of media and the same incubation treatment incubated with and without SC ($p \leq 0.05$).

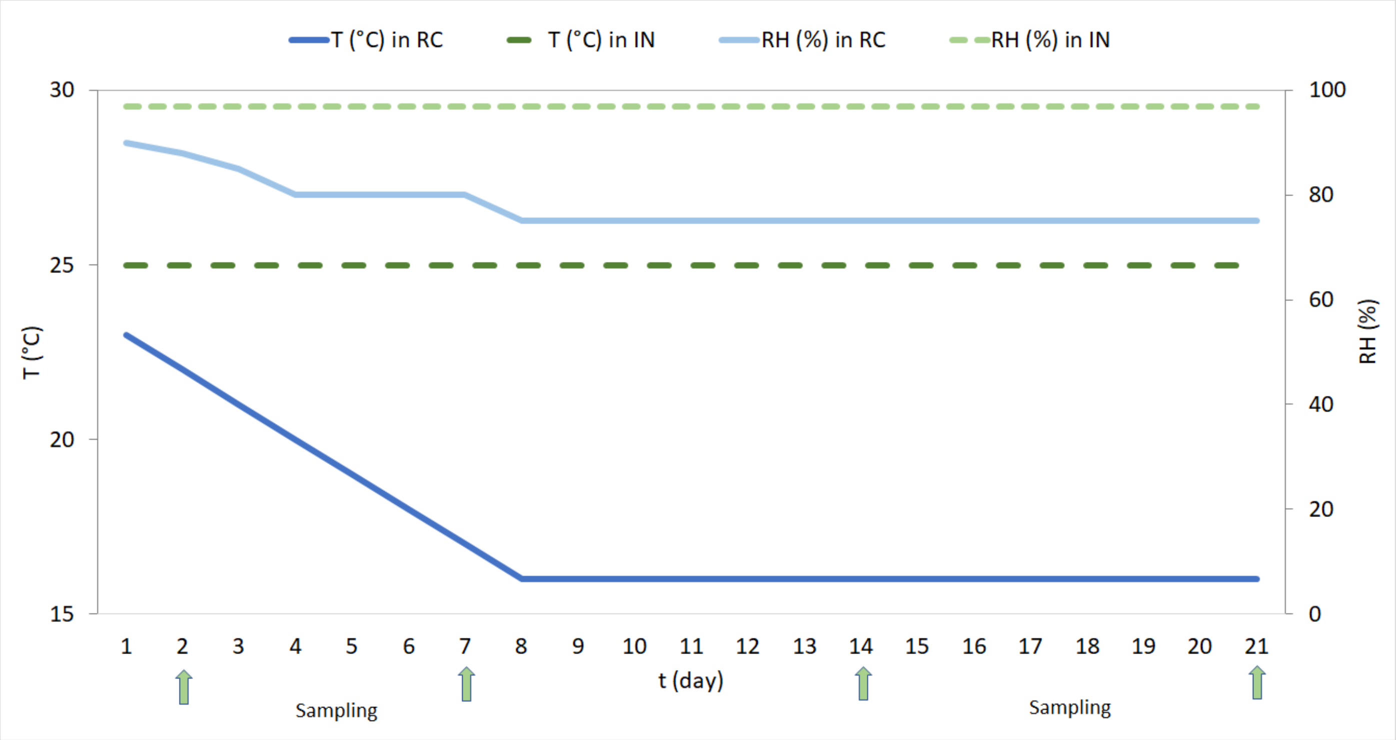


**Figure S1**. Temperature and relative humidity during the 21-day incubation of media in the ripening chamber (RC) and in the incubator (IN).
